# SELEX: How It Happened and Where It will Go

**DOI:** 10.1007/s00239-015-9705-9

**Published:** 2015-10-20

**Authors:** Larry Gold

**Affiliations:** SomaLogic and the University of Colorado, Boulder, CO USA

## The Chance to Contribute to This Special Issue

I feel privileged to be asked to write something for this collection of articles about aptamers. I was also flattered: 25 years is a long time to try to stay relevant! I decided to break this short article into “First Moments,” the “NeXstar Moment,” the “SomaLogic Moment,” and “Next Moments.” By the section names alone, I hope the reader will recognize a life lived with intense focus, the short-sightedness of seeing the world through my own eyes, but also a life lived reading the literature broadly. Without concern, I did not cite many papers in this article because most are old but well known; however, I am happy to provide references to anyone who asks for them (email at lgold@somalogic.com).

## The First Moments

Craig Tuerk, a remarkable PhD student in Molecular, Cellular, and Developmental Biology at the University of Colorado in Boulder (and still a remarkable person, currently a Professor of Biology at Morehead State University), was finishing his thesis on the translational regulation of the DNA polymerase gene of the bacteriophage T4. The “operator” on that mRNA was a binding site for the polymerase, which thus repressed its own translation when the polymerase level was high enough. That RNA sequence/structure overlapped the ribosome binding site, and was the piece of RNA that Craig studied. A hairpin, with an intramolecular loop of eight nucleotides, was within the operator and the loop became the subject of Craig’s deep mutagenesis, in which every possible loop sequence (all 4^8^ of them) was selected for binding to the polymerase. Two sequences emerged (the wild-type and the major variant, containing four nucleotide changes within those eight loop nucleotides) and Craig and I shared the most wonderful moment possible for scientists: we imagined a future in which RNAs were “shapes, not tapes” or “strings, not things” and were useful in the same way that monoclonal antibodies are useful. Craig named the process “SELEX” (Systematic Evolution of Ligands by EXponential enrichment) in his *Science* paper (Tuerk and Gold 1990), while Andy Ellington and Jack Szostak named the resulting molecules “aptamers” in their *Nature* paper (Ellington and Szostak 1990). Both words have stuck—one “does SELEX” and “gets aptamers.”

Neither Andy and Jack nor Craig and I were the first to do in vitro mutagenesis on this scale. Arnold Oliphant and Kevin Struhl had published a beautiful paper in 1989; they synthesized double-stranded DNA with 25 base pairs of fully random sequence (4^25^ sequences) and selected those sequences that bound to the yeast transcription factor GCN4 (Oliphant and Struhl 1989). That paper did not utilize “rounds” of amplification and selection as SELEX does, but PCR made that addition to the Oliphant/Struhl procedure sensible; Oliphant and Struhl merely made a lot of double-stranded DNA and used affinity chromatography (two “rounds” without amplification between the rounds) to pull out the right sequences.

The major difference between working on double-stranded and single-stranded oligonucleotides was huge, we thought. Helices (of RNA or DNA) do not have, usually, fascinating shapes, while single-strands, with some intramolecular Watson–Crick helices and lots more, really are (or so we thought) like the antigen-binding sites of monoclonal antibodies. We saw single-stranded oligonucleotides as globular molecules, much like proteins. (All of the work in our lab for almost two decades prior to this moment considered mRNA to have functional intramolecular structures, so we were “ready” for our discovery.) For several days in a row, Craig and I dreamed about monoclonal antibodies made out of nucleic acids. The first scientist Craig and I talked to outside of our lab was Ed Brody (then in Paris), who did not dump on our idea; in fact, in 1999, he became part of the NeXstar-SomaLogic team. Craig and I talked to a lot of scientists in Boulder and elsewhere as we were writing the paper, and Ed was one who thought it was sensible and generalizable—most of our friends told us (gently, usually) that we were being silly, even though we all knew by then about Tom Cech’s group I intron’s catalytic activity.

The last 25 years have made the idea of “antibodies” made from single-stranded oligonucleotides a reality. Today, because of the 10,000 or so papers about natural and synthetic and aptamers and SOMAmer reagents, and probably as many papers about ribozymes, riboswitches and DNAzymes, it is easy to think that we thought about oligonucleotides as shapes in the early 1990’s. In fact, we did think of RNA as a shape if and only if one was thinking about 5S RNA, rRNA, a group I intron, RNase P, or a tRNA—that is, if one was thinking about molecules that were thought to be outliers! Even today, text books written by professionals show mRNA as a random coil—as though base-pairing was not inevitable for single-stranded oligonucleotides—I have often called this the “tyranny of *Watson*-*Crickery*.” The wonderful thing that Craig and Andy made more likely was the generalization that there were huge numbers of shape possibilities in single-stranded oligonucleotides, and the SELEX protocol was going to identify them.

## The NeXstar Moment

We started NeXagen in 1992. NeXagen became NeXstar when we merged with Vestar, and then, we sold the whole thing to Gilead in mid-1999. During those 7 years, we did a lot of development of methods to expand how we thought about and performed SELEX. The major accomplishments at NeXstar centered on Macugen, the first VEGF antagonist approved for use in age-related macular degeneration (AMD), and also on the PDGF aptamer antagonist that is today undergoing clinical development by Ophthotech (this compound is also for AMD, and appears—as Nebojsa Janjic predicted almost 20 years ago—that inhibiting PDGF and VEGF would synergize for diseases characterized by abundant and inappropriate angiogenesis, including that process in the “wet” form of AMD). Nebojsa was the major scientist at NeXstar (among many great scientists), and both Macugen and the PDGF antagonist were identified in his group.

Bruce Eaton joined NeXagen early, and brought with him methods for adding modifications to the five position of the pyrimidines (see below), as well as doing early work on the larger scale synthesis of aptamers. We also did cell-SELEX, covalent-SELEX, photo-SELEX, and many other additions to the breadth of SELEX; at one point, NeXstar scientists published a remarkable selection aimed at a small molecule—theophylline—which was nearly identical to caffeine (which has a methyl instead of a hydrogen at a single position); in that work, an RNA aptamer was identified that bound to theophylline about 10^4^ more tightly than to caffeine! At that time, antibodies raised against theophylline were not nearly that selective against caffeine.

## The SomaLogic Moment

When NeXstar was sold, many of the scientists moved around the world into other biotech and pharma companies. Nebojsa became the co-founder of an antibiotic discovery and development company. Bruce started a company aimed at using modified RNAs to do catalysis to identify orally active drugs. Some NeXstar scientists were willing to join me in 2000 to create SomaLogic, which was aimed at developing diagnostics. Bruce and I were able separately to negotiate the purchase of the required “NeXstar” IP from Gilead (who was fair and helpful). Gilead knew what it wanted from the NeXstar purchase and made it easy for Bruce and me and, later, the founders of Archemix, to continue our dreams.

SomaLogic was started around a simple idea that we had said out loud at NeXstar in 1997. We knew that people were going to crush *genomics* (which turned out to be right, of course), and we knew that *proteomics* was going to be far more difficult. We also believed (and I continue to believe) that medical diagnostics was not as useful for patients and healthcare as it had to be, and that *personalized medicine* would depend on *genomics* and *proteomics* (and other *omics* technologies). Before NeXstar was sold, we thought we understood how to quantify thousands of proteins using aptamers.

Because we were biochemists at heart (some of us had become biochemists when fields like molecular biology did not exist!), we fully understood that antibodies would continue to be used for single analytes: ELISAs are a great invention, and sandwich assays allow two elements of specificity to generate enormous specificity! However, we also understood, through some modeling that Dom Zichi did on the back of an envelope, that arrays of sandwich ELISAs were going to reach quickly a content size that would compromise signal-to-noise in the measurements. So we set out to create arrays of aptamers that would allow thousands of proteins to be measured at once, thus making protein biomarker discovery as simple a process as mRNA, microRNA, or SNP biomarker discovery is today.

To eliminate the intrinsic noise of sandwiches at high density (which we have described in several publications), we needed to select aptamers with two elements of specificity in a single molecule (a kind of “intramolecular sandwich”). We fiddled with coupling photo-crosslinking along with high specificity binding, but moved rapidly (but not as rapidly as we might have had I not been so stubborn) toward a combination of kinetics with high specificity binding. As we have written earlier, we were comforted by the work of John Hopfield on “kinetic proof-reading” in the mid-1970s, and understood from his work that kinetics could be used to enhance specificity.

At that moment, more or less, we realized that the earlier work at NeXstar by Bruce and his co-workers (on those pyrimidine adducts that make oligonucleotides more chemically diverse) was going to make the task of high specificity-high affinity-slow off-rate binding a lot easier, and we have extended Bruce’s work enormously. Medicinal chemists at SomaLogic, notably John Rohloff (with Nebojsa, who has come back to his true home, and a team of several additional chemists) have created a large set of pyrimidine adducts, all sitting on the *privileged* five position of Cytosine and Thymine; by *privileged* I mean only that adducts at that position are accepted by many DNA polymerases (both as templates for PCR and as triphosphates substrates)—the critical amplifications required for rounds of SELEX could still be done.

During the last few years, the scientists at SomaLogic have used thousands of proteins (mostly human, some other mammals, and some bacterial) as SELEX targets, using a collection of modified pyrimidines (with full substitution for any individual selection, to avoid a difficult deconvolution). The four X-ray structures of complexes between these novel aptamers and their target proteins solved to date (three of these are published) support fully that single-stranded oligonucleotides can be globular molecules, much like proteins; the new “side chains” function in these new aptamers as do many different amino acid side chains in proteins. What Bruce once called “side chain envy” is no longer a problem; these new reagents have the folding and target-contact opportunities of both single-stranded oligonucleotides and proteins.

We have called these new reagents “SOMAmers” (Slow Off-rate Modified Aptamers). We believe that additional chemistries are more important to identifying great aptamers than, for example, additional nucleotides (beyond the canonical four) that can independently base pair without confusing the canonical four. Additional base pairs are interesting to me as a way of wondering about early evolution. (My mentor Carl Woese did not have much time to consider the question “why four nucleotides?”—surely he would have said something smart, since he always did that.)

The primary use of the collection of SOMAmer reagents has been to quantify proteins in biological matrices, using a large (and expanding) multiplex platform called the “SOMAscan” assay. Biomarkers have been found robustly, quickly, and reproducibly, using small amount of precious samples, by SomaLogic and many academic and pharma/biotech collaborators using this remarkable new assay. Once found, protein biomarkers can be used in all the common medical uses of protein detection (now done largely with ELISAs or mass spectrometry), but with the added capability of designing and implementing panels of measurements aimed at particular medical problems. A favorite paper in my life is the recent paper about Duchenne Muscular Dystrophy, published in *PNAS* with many authors (Hathout et al. 2015), and driven largely by Pat Furlong and Fintan Steele (an old friend who works at SomaLogic). One of the joys of having the SOMAscan assay running in our labs is that we get to collaborate with wonderful people.

## The Next Moments

I think of SOMAmer reagents (and, in fact, all aptamers) as exact homologues of the antigen-binding domains of antibodies, except that they are made of nucleic acids and thus produced synthetically. As such it is trivial to functionalize aptamers with anything that can be prepared as a phosphoramidite, and almost anything can be prepared that way. This means that the future for applications of aptamers will be limited only by our imaginations, as is always the case. Already aptamers have been used for proteomics, cell sorting, pathology, affinity purification, and pharmaceuticals, and those are just the things that build on prior work with antibodies.

I suspect that the many inventions to come will be based on novel chemistries added to aptamers, as we have done, but not limited to what we have done. Our focus has been *proteomics* (for nearly two decades), but what if people begin to address nanotechnology in all its (unexplored) domains? Novel aptamers have beguiling qualities: chemical synthesis, high affinity for one or more targets (large or small), exquisite shape, acceptable resistance to things in the environment, and uses we certainly have not said out loud. I love the idea of material scientists imagining the properties they want in a small oligonucleotide, and further imagining adding their stuff to those privileged five positions. Molecular origami will benefit from modified pyrimidines and SELEX, as will nano-electrical devices—SOMAmer reagents provide an opportunity to place in space, in those beautiful globular entities, whatever one imagines will be useful for any structural and functional purposes (with binding being the earliest and easiest thing to do). I look forward to the next 25 years for this field, and expect to write another review on my 100th birthday.
